# A novel strategy for dynamic identification in AC/DC microgrids based on ARX and Petri Nets

**DOI:** 10.1016/j.heliyon.2020.e03559

**Published:** 2020-03-27

**Authors:** Leony Ortiz, Luis B. Gutiérrez, Jorge W. González, Alexander Águila

**Affiliations:** a Carrera de Electricidad, GIREI, Universidad Politécnica Salesiana, Quito, Ecuador; b Grupo de Investigación Aeroespacial, Universidad Pontificia Bolivariana, Medellín, Colombia; c Grupo de Investigación en Transmisión y Distribución, Universidad Pontificia Bolivariana, Medellín, Colombia

**Keywords:** Energy, Identification, Microgrid, No-linear systems, Petri net, State space model, ARX

## Abstract

This paper presents a new hybrid strategy which allows the dynamic identification of AC/DC microgrids (MG) by using algorithms such as Auto-Regressive with exogenous inputs (ARX) and Petri Nets (PN). The proposed strategy demonstrated in this study serves to obtain a dynamic model of the DC MG in isolated or connected modes. Given the non-linear nature of the system under study, the methodology divides the whole system in a bank of linearized models at different stable operating points, coordinated by a PN state machine. The bank of models obtained in state space, together with an adequate selection of models, can capture and reflect the non-linear dynamic properties of the AD/DC MGs and the different systems that it composes. The performance of the proposed algorithm has been tested using the Matlab/Simulink simulation platform.

## 1 Introduction

Thanks to recent technological advances, the growing demand for energy has caused the load on the transmission network to increase at an accelerated rate. From an economic point of view, updating the transmission grid is difficult due to its high cost, that is why MGs have become an economically viable alternative. The MGs that complement the Electric Power System (EPS) offer benefits from a system resilience view: loads are provided with energy even if the transmission grid is inactive due to a failure.

Studies such as [1,2], which seek to solve problems related to the resilience of the EPS have undoubtedly opted for the implementation of smart microgrids (SMG). These studies also address that microgrids will play an important role in the new decentralized paradigm of smart grids (SG). Certainly, MGs are a path to the evolution of SGs, progressively becoming ideal prototypes both for isolated sites and sites interconnected to the national electrification system [1,2].

In order to develop fault tolerant control strategies for a MG, and to investigate the stability of small signals when fault conditions are present, where most of the methods are based on knowledge of the normal behavior of the system, an adequate model of the MG and its components is necessary. This explains why for the scientific community, a new subject of great interest is to obtain these mathematical models from the MG [3–5].

However, the identification of systems with unknown dynamic characteristics that are difficult to capture, or highly non-linear ones have opened a highly important field of research entitled, "*system identification*". Basically, the identification of systems is a set of statistical and stochastic methods that allows to find a mathematical model suitable for a known dynamic system. This mathematical model is adjusted with the use of observed data of the unknown system [3,6].

In this paper, a novel strategy is developed through the use of PN and ARX that allows us to identify a bank of dynamic models in state space (considering the system as "black box") that describe the evolution of the different components of the MG towards the steady state. In addition, the model developed is suitable for the fault tolerant control design using the nominal values of voltage and current of the inverters and converters. The model is also useful for the evaluation and improvement of small signal stability.

To validate the results of this research study, a hybrid microgrid (HMG) benchmark [7] was considered constituted with load bus and generation distributed at medium and low voltage levels in AC and a DC bus. The calculations corresponding to the results obtained from all the variables analyzed for this investigation were made using algorithms implemented in Matlab environment. Additionally, the detailed simulation model is based on the Simulink graphical programming environment.

The organization of the paper is as follows: Section 2 “*Electric Microgrid*” presents the main characteristics of the MG modeling and the MG architectures. Section 3 “*Petri Net*” and IV ‘*Identification problem*’ provides a brief description of the PN and ARX identification algorithms used in the investigation. Section 5 discusses the analysis of the identification problem and the proposed strategy. Section 6 describes the simulation results of the proposed dynamic identification strategy and finally, the future research areas and conclusions can be found in Section 7.

## 2 Electric microgrid

In the future, MGs are seen as an attractive solution for the integration of Distributed Generator (DG) units in the SG, which will allow less dependence on fossil fuels and an increase in the efficiency of the distribution systems. However, the challenges for MGs are greater due to: fast dynamics and short response time of DG, inherent unbalanced nature of the MG, low energy storage capacity and lack of inertia, a high number and diversity of micro-sources, electronic power converters and other circuits/devices, high degree of parametric uncertainties, modeling, and high failure rates [7,8].

**Figure 1:**
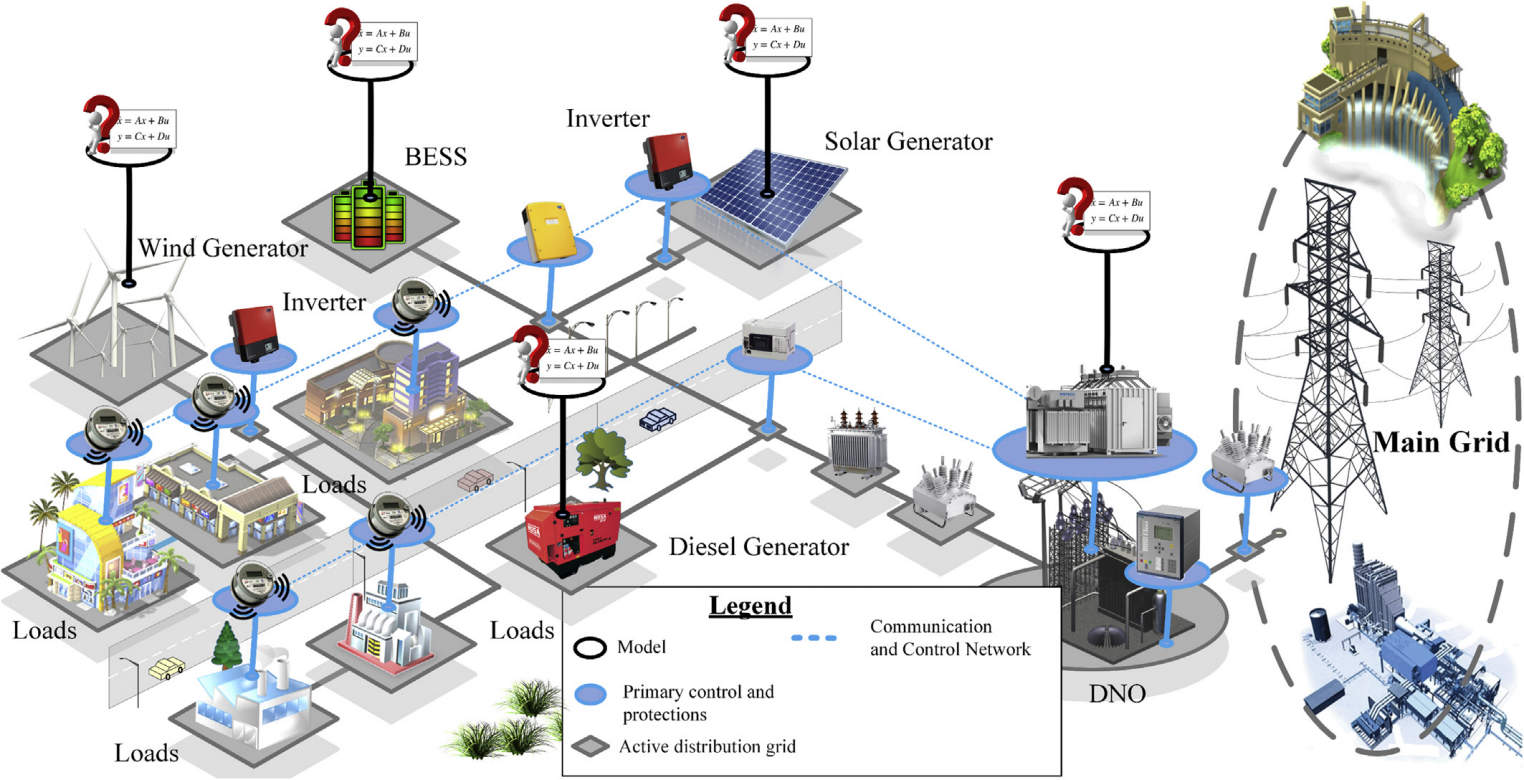
Identification problem of microgrids.

Figure 1 shows an architecture constituted by a series of systems and subsystems such as: distributed generation, energy storage and loads.

MGs must be robust to control the voltage, frequency and protect the main grid and the loads connected to the various faults [8,9,10]. MGs also facilitate the administration and re-synchronization of the demand side. There are some types of SGs on a small scale that provide one of the most interesting solutions to improve power flow in distribution grids and reduce power losses in transmission lines [11,12,13,14,15].

### 2.1 Microgrid architecture

In the literature, three main MG definitions can be found: MG, nano-grids (NG), and pico-grids (PG). Additionally, MGs can operate in parallel to the main grid, both in stand-alone mode (stand-alone power) and in interconnected mode (assumes main grid instructions) [7,8,12,15,16,17,18,19,20,21,22,23]. The most used MGs are usually classified according to their different types:

• Type of voltage (DC, AC, hybrid),
• Type of distribution (single-phase, three-phase, three-phase + neutral),
• Type of voltage (low (LV), Medium (MV)),
• Structural (radial, ring).

MGs that can be implemented are in the Series, Switched and Parallel configurations as described in [24]. In the MG series configuration there is a DC bus where all the generation systems and loads are connected through their respective converters. On the other hand, the parallel configuration has an AC bus where the generation systems and the loads are directly connected. Additionally, the DC devices are connected through their own inverters, or by a DC bus coupled to the AC bus which are connected using an inverter or bidirectional voltage-sourced converter (VSC) [7,8,24].

**Figure 2:**
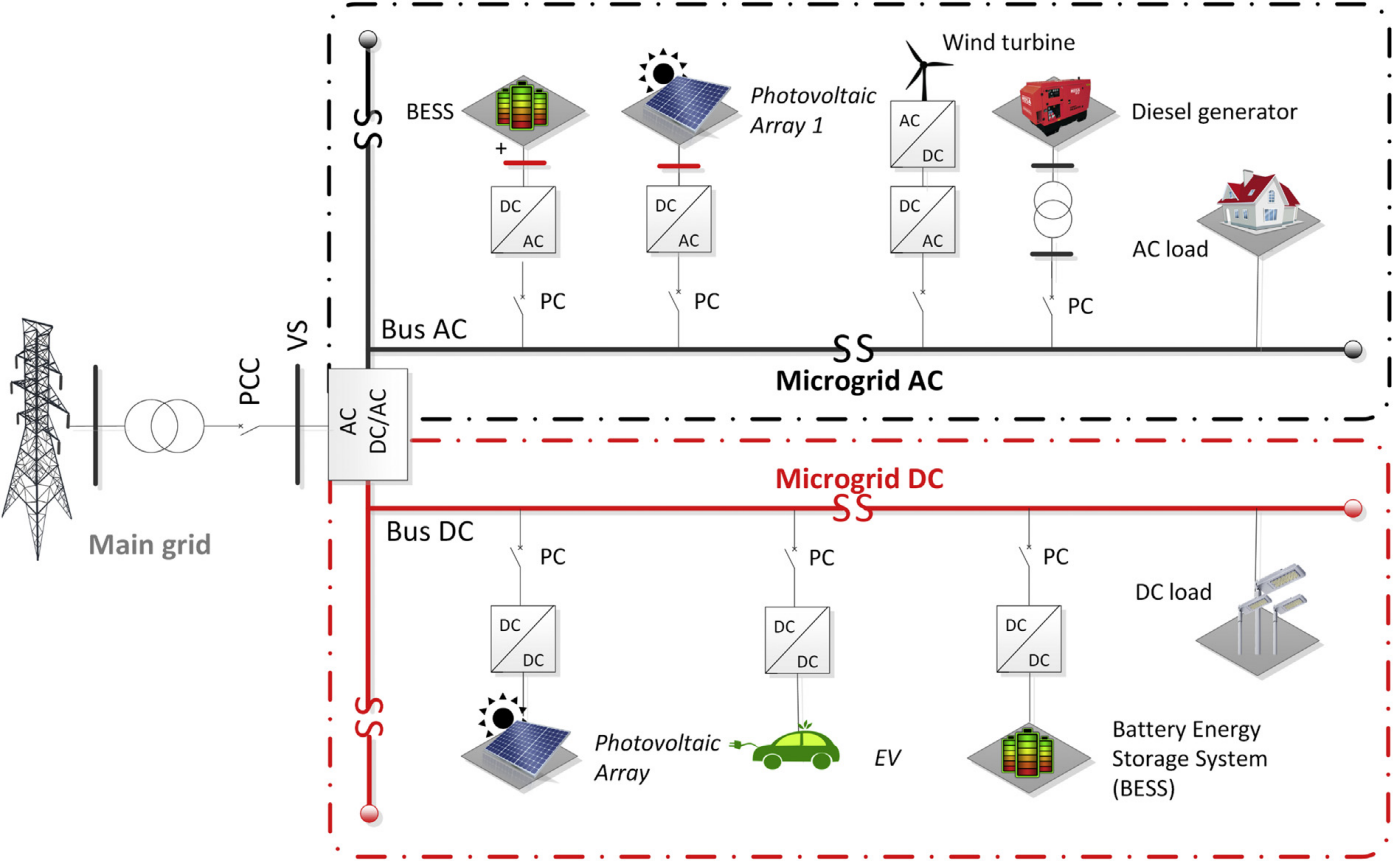
AC/DC hybrid microgrid.

The combination between the AC and DC MG configurations has resulted in the concept of AC/DC HMGs (see Figure 2) and proposes a better approach because it combines the main advantages of the AC and DC MGs [10]. Future trends show that features such as scalability, modeling, design, and control structures require further investigation to achieve integration of HMGs into the main grid [7,15,23].

AC architectures can be seen from two approaches: interconnected or coupled MGs and isolated or decoupled MGs. In turn, two approaches have been identified in coupled MGs: fully and partly isolated topology [7,24].

On the other hand, decoupled AC MGs display three topologies which are the following: the two-stage total isolated configuration, the two partially isolated stages, and, finally, the configuration of three partially isolated stages. The configuration to be defined for the AC/DC HMG depends greatly on the application and the environment in which it is included, for example: energy market conditions, regulatory conditions, feasibility for the integration of DG units, among others [7,12,15,20,23,25].

### 2.2 Microgrid modeling

By understanding the dynamics of the different systems and subsystems used in the MGs, it is important to implement methodologies of modeling and identification. These methodologies are vitally important to carry out an accurate analysis of the control systems or the design of fault tolerant control systems (FTCS). Although MGs are considered small scale, their complexity is extremely high compared to the conventional energy system. Therefore, its dynamic analysis is a key point that ensures that the controllers for the MGs work in a stable and reliable way before controlled fluctuations of voltage and other variables. In this context, simulations are a tool able to closely resemble, some of the properties and the behavior of MGs. These, when applied to MGs, imply a significant saving of money and time.

The models of the MGs are dynamic and can change depending on their configuration, type of topology, and components. Therefore, different modeling methodologies are required that are capable of representing important phenomena: rapid dynamics and short response time of DG, inherent unbalanced nature of the MG, low energy storage capacity and lack of inertia, a high number and diversity of micro-sources used, electronic power converters and other circuits/devices, high degree of parametric uncertainties, modeling, and high failure rates [10,11,15,26,27,28,29].

In the dynamic modeling, the very slow and fast dynamics are often neglected. For example, the dynamics of generator (conventional sources) are much slower than the dynamics of VSC, which can disregard the generator dynamics. Many authors consider it as a constant parameter to the voltage on the generator bus and not model the associated dynamics. In addition, due to the high switching frequency of some elements like converters/inverters, the relevant dynamics are also considered insignificant [5,8].

On the other hand, authors seek to model each source of DG reducing the order of the model to a first order and linear time-invariant (LTI) with a time constant and gain factor, but neglect the dynamics of the grid. Other researchers represent the MG by a DC source with a VSC connected to the main grid, an RL filter, a transformer and a circuit breaker. In this way it is simple to obtain a low order dynamic model for the equivalent system that can be easily used for the purpose of control analysis [5,8].

There are also other approaches with DG based on inverters, for example in [30] the complete dynamic model of the entire grid was considered in place of the inverter, dividing the MG system into three subsystems: inverter, distribution grids, and loads. In the inverter model, the dynamics of the controller, the output filter and the coupling inductor are incorporated. Additionally, the state equations of the grid and the loads are represented in one of the reference frames of the inverter, assuming that this is the common reference. Then, using the transformation technique, the other inverters are transformed into this common framework and model each subsystem in combined state-space in the aforementioned common frame of reference. In [31], authors also develop a model in LTI state space and perform an analysis of the eigenvalues to study the dynamic behavior of the MG, while the electrical parameters and control gains are modified.

Furthermore, additional articles as in [32] propose a model using the nominal values of the inverter frequency and voltage set point (VSI) and the active and reactive power set points of the PQ inverters. This type of dynamic model based on state space is useful to evaluate and improve the stability of small signals, design and implement distributed and centralized secondary controllers, taking into account storage device characteristics.

## 3 Petri net

PNs allow the study and description of information processing systems characterized by being concurrent, distributed, non-deterministic and/or stochastic [33,34,35,36]. A PN is a rigorous and powerful mathematical and graphic representation, commonly used in discrete, distributed and continuous systems [33,36,37,38,39].

### 3.1 Formal definition of a Petri Net

Considering a PN as 5-tuple, PN = (P, T, F, W, Mo) where [33,37,40,41]:

- $P=\left\{p_{1},p_{2},\ldots p_{m}\right\}$ is a finite set of places,
- $T=\left\{t_{1},t_{2},\ldots t_{r}\right\}$ is a finite set of transitions,
- F⊆(PxT)∪(TxP) is a set of arcs,
- W:F→{1,2,3,4…} is a weight function,
- $M_{0}:P\to N:\left\{0,1,2,3,4\ldots \right\}$ is the initial marking,

-P∩T=ϕ and P∪T=ϕ

- I:PxT→N is the input function that defines directed arcs from places to transitions, where *N* is a set of positive integers.
- O:TxP→N is the output function that defines directed arcs transitions to places.

PNs can be represented in matrix form, it is called incidence matrix (*C*) whose dimension is defined by the number of places (*P*) represented by the rows and number of transitions (*T*) corresponding to the columns. The matrix *C* is defined by:

(1) $$C\left(P,T\right)=\;\{\begin{array}{cc} W^{-}\left(p,t\right) & iff\left(p,t\right)\in F\\ W^{+}\left(p,t\right) & iff\left(t,p\right)\in F\\ 0 & otherwise \end{array}$$

*F* is the flow ratio between *P* and *T, W* (*p*, *t*) is the arc weight between *P* and *T.* Furthermore*, f* (*p, t*) ∈ *F* means that there is no flow ratio from *P* to *T* and the incidence matrix of a PN is given by:

(2) $$C\left(p,\;t\right)=W^{+}\left(p,\;t\right)-W^{-}\left(p,\;t\right)$$

where *W* ^*+*^(*p*, *t*) is the incidence matrix input and *W* ^*-*^ (*t*, *p*) is the incidence matrix output.

Matrix representation of PN:(3)
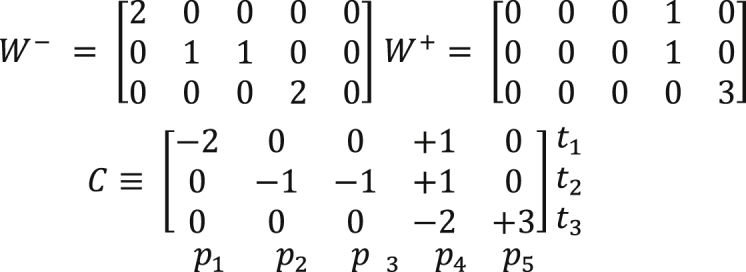


Incidence Matrix provides information on the final balance in the number of brands that occur in places of a PN when the corresponding transitions are shot.

### 3.2 Basic structure of a PN

**Figure 3:**
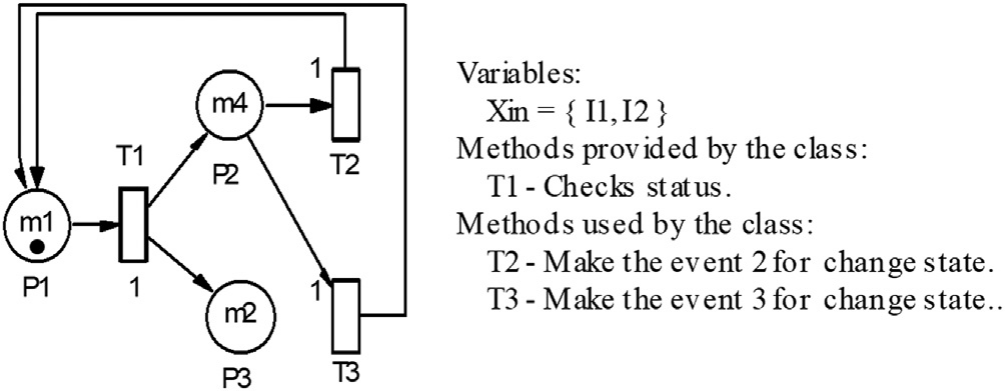
Petri net [33].

The PNs are a directed graph that contain two types of main nodes: the places (represented by circles) and the transitions (represented by rectangular bars), together with one or several initial states that is called the initial marking (represented by tokens or black dots) located within each place as shown in Figure 3 [33,36,38,42]. The PN of this figure consists of three places, seven transitions, one token and seven arcs of unit one weight [33,37,39].

The nodes are interconnected with directed arcs, which connect the transitions with places and vice versa, in addition to modifying the different states of the system according to the assigned weights. The weight of an arc determines the number of marks that the place consumes or that is deposited in another place, as long as the enabled transitions are triggered. Arcs directed without a number are understood to consume or deposit a mark [28,33,34,36,38,42].

Therefore, the PN of Figure 3 can be written mathematically in the following:

$$P=\;\left\{p_{1}\;,\;p_{2}\;,\;p_{3}\right\}\;;T=\;\left\{t_{1}\;,\;t_{2\;}\;,\;t_{3}\right\}\;;I=\;\left(p_{1}\;,\;\;t_{1}\right)=1\;$$

$$I=\;\left(p_{2}\;,\;\;t_{2}\right)=1\;;\;I=\;\left(p_{2}\;,\;\;t_{3}\right)=1\;;\;I=\;\left(p_{2}\;,\;\;t_{2}\right)=1\;$$

$$I=\;\left(p_{3}\;,\;\;t_{2}\right)=0\;;\;I=\;\left(p_{3}\;,\;\;t_{3}\right)=0\;;\;I=\;\left(p_{3}\;,\;\;t_{i}\right)=\;0$$

$$O=\left(t_{1}\;,\;\;P_{2}\right)=1\;;\;O=\left(t_{1}\;,\;\;P_{3}\right)=1\;;\;O=\left(t_{2}\;,\;\;P_{1}\right)=1\;\;$$

$$O=\left(t_{3}\;,\;\;P_{1}\right)=1\;;\;O=\left(t_{i}\;,\;\;P_{j}\right)=0\;;\;M_{0}={\left(1,\;0,\;0\right)}^{T}\;\;$$

$$O=\left(t_{3}\;,\;\;P_{1}\right)=1\;;\;O=\left(t_{i}\;,\;\;P_{j}\right)=0\;;\;M_{0}={\left(1,\;0,\;0\right)}^{T}\;;\;$$

## 4 Identification problem

The identification of dynamic plants depends to a large extent on the different families of possible models and their stochastic environments. It also depends on the correct choice of the selection criteria of the model classes [6,43]. The problem of the identification of systems deals with the estimation of dynamic system models from observed data of inputs *u* (*t*) and outputs *y* (*t*), managing the system as a black box operating in a stochastic environment [6,43].

For the systems identification, the most studied methods are based on the step response such as: the Oldenbourg - Sartorius method; Anderson's method; step response for oscillatory systems; method Strejc for systems of order *n*; models with transport delay; among others [4,6,43].

**Figure 4:**
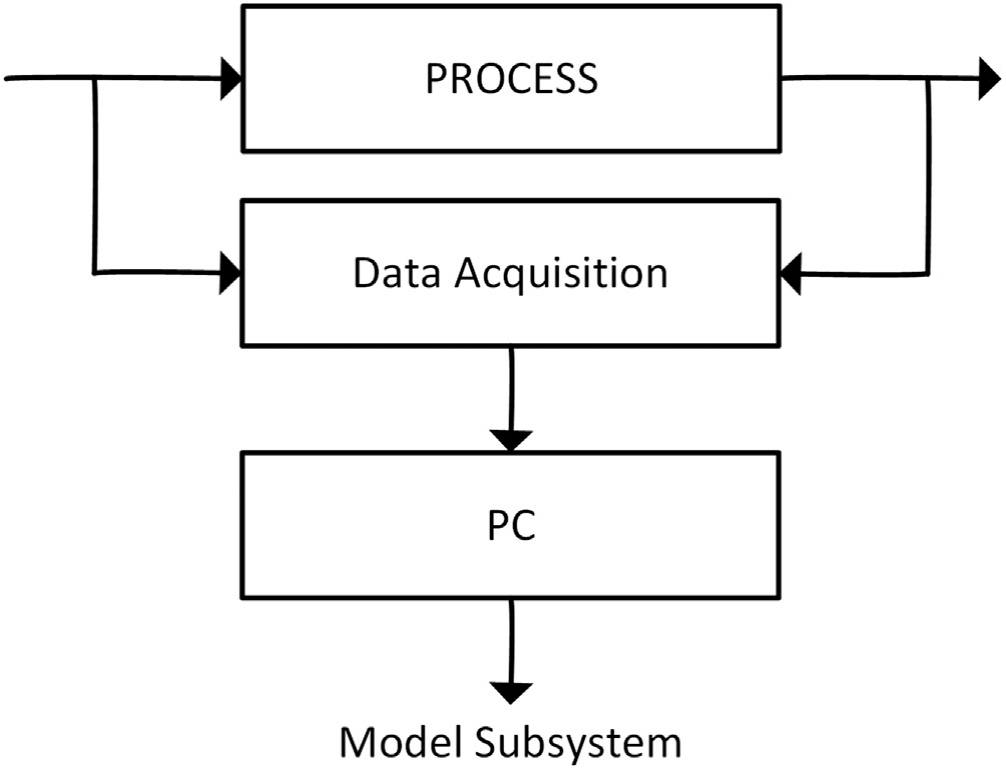
Identification model.

Another point to make in the identification problem and the scope of control theory, is the importance to bear in mind that it is not the same to identify a model for a plant (controlled system - MG) that will work in open loop or closed loop. Open-loop operation will obviously require greater precision and less uncertainty [4,43]. Figure 4 shows the identification model.

Designing strategies to obtain dynamic models based on identification provides the complement for detection and diagnosis of failures that provide solutions based on the use of state models [44].

### 4.1 Model in the state space

The state space is an *N*-dimensional space which consists of the axis *x*_*1*_, axis *x*_*2*_, ...... axis *x*_*n*_. Any state can be represented by a point in the state space [32,45].

Representation of the state model:

(4) $$\dot{x}\left(t\right)=\left[\begin{array}{c} {\dot{x}}_{1}\left(t\right)\\ {\dot{x}}_{2}\left(t\right)\\ \vdots \\ {\dot{x}}_{n}\left(t\right) \end{array}\right]\;\;=\;f\left(x,u,t\right)=\left[\begin{array}{c} f_{1}\left(x_{1},x_{2},\cdots ,x_{n};u_{1},u_{2},\cdots ,u_{r};t\right)\\ \vdots \\ f_{n}\left(x_{1},x_{2},\cdots ,x_{n};u_{1},u_{2},\cdots ,u_{r};t\right) \end{array}\right]$$

(5) $$y\left(t\right)=\left[\begin{array}{c} y_{1}\left(t\right)\\ y_{2}\left(t\right)\\ \vdots \\ y_{m}\left(t\right) \end{array}\right]\;\;=\;g\left(x,u,t\right)=\left[\begin{array}{c} g_{1}\left(x_{1},x_{2},\cdots ,x_{n};u_{1},u_{2},\cdots ,u_{r};t\right)\\ \vdots \\ g_{m}\left(x_{1},x_{2},\cdots ,x_{n};u_{1},u_{2},\cdots ,u_{r};t\right) \end{array}\right]$$

Linearizing Eqs. (4) and (5) around the operation state:

(6) $$\dot{x}\;\left(t\right)=\;A\left(t\right)\;x\left(t\right)\;+\;B\left(t\right)\;u\left(\text{t}\right)$$

(7) y(t)=C(t)x(t)+D(t)u(t)

where:

- *A* (*t*) ∈ R^nxn^ is the state matrix,
- *B* (*t*) ∈ R^nxr^ is the excitation matrix,
- *C* (*t*) ∈ R^mxn^ is the output matrix,
- *D* (*t*) ∈ R^mxr^ is the direct transmission matrix,
- *x* (*t*) ∈ R^nx1^ is the state vector,
- *u* (*t*) ∈ R^rx1^ is the input vector,
- and *y* (*t*) ∈ R^mx1^ is the output vector.

### 4.2 Auto-Regressive with eXogenous inputs

The models identified for plants that represent dynamic systems depend on the families of models considered, capable of describing different stochastic environments. The criteria for selecting a model within a specific class of models is based primarily on the model being able to predict the observed dynamic behavior rather than adherence to an associated stochastic context. This happens because the systems are actually much more complex than the representations used for their description. The ARX is “*Auto-Regressive with eXogenous inputs*” model structure. The ARX allows to represent a dynamic process modified by one or more input entries considering the uncertainties. This model describes the observed *y* (*t*) output of the system, process or plant as a regression sum of the observations of the inputs *u* (*t*) and output *y* (*t*) and the error *e* (*t*) of the model [6,43].

Considering input and output data:

(8) $${\left\{\left(u\;\left(t\right),\;y\;\left(t\right)\right)\right\}}_{t=1}^{N}.$$

The linear ARX model is represented by the following equation:

(9) $$y\left(t\right)+a_{1}y\left(t-1\right)+\ldots +a_{na}y\left(t-na\right)=b_{1}u\left(t-nk\right)+...+b_{2}u\left(t-nk-1\right)+\ldots +b_{nb}u\left(t-nk-nb+1\right)+e\left(t\right)$$

The dynamic system output from data and disturbances is given by:

(10) $$y\left(t\right)=-a_{1}y\left(t-1\right)-\ldots -a_{na}y\left(t-na\right)+b_{1}u\left(t-nk\right)+...+b_{2}u\left(t-nk-1\right)+\ldots +b_{nb}u\left(t-nk-nb+1\right)+e\left(t\right)$$

A lineal regression point is described by:

(11) $$\theta \;=\;{\left[a_{1}\;...\;\;a_{na}\;\;\;b_{1}\;...\;b_{nb}\right]}^{T}$$

(12) $$\varphi \left(t\right)={\left[-y\left(t-1\right)\;...\;-\;y\left(t-n\right)\;u\left(t-1\right)\;...\;\;u\left(t-m\right)\right]}^{T}$$

(13) $$y\left(t\right)=\varphi^{T}\left(t\right)\;\theta$$

The output prediction is given by:

(14) $$\hat{y}\left(t|\theta \right)=\varphi^{T}\left(t\right)\theta |$$

The natural definition of the prediction error is:

(15) $$\omega \;\left(t\;\left|\;\theta \right)\;=\;y\left(t\right)\;-\;\hat{y}\;\left(t\;\right|\;\theta \right)\;\;$$

Under the assumption that *e*(*t*) is white noise, the least squares method is used to help minimize the sum of the squares of the right member of the equation with respect to the coefficients *a* and *b.* This generates a linear system of equations based on the measurement of the instrumental variables where *a* and *b* are the unknowns.

## 5 Statement of the problem

Due to the widespread use of commercial DG sources and other factors, it is laborious to build a model for the HMG by mechanism modeling. However, if a dynamic identification method automatically coordinated by a state machine is developed, while considering the HMG as a bank of linearized models at each permissible operating point, each element of the HMG may be seen as a black box system with multiple-input and multiple-output (MIMO).

Considering an AC/DC HMG, both in its connected mode to the grid and in its isolated mode composed of: distributed energy resource (DER) and a set of smart systems designed for the conversion of energy (DC/DC, DC/AC, AC/AC, bidirectional or not), control, monitoring and protection. The objective of this paper is to obtain a model in the state space of the whole MG for control purposes using the observed information, where the very fast dynamics of highly non-linear systems such as inverters and rectifiers are not the main objective for the design and tuning of the controllers.

**Figure 5:**
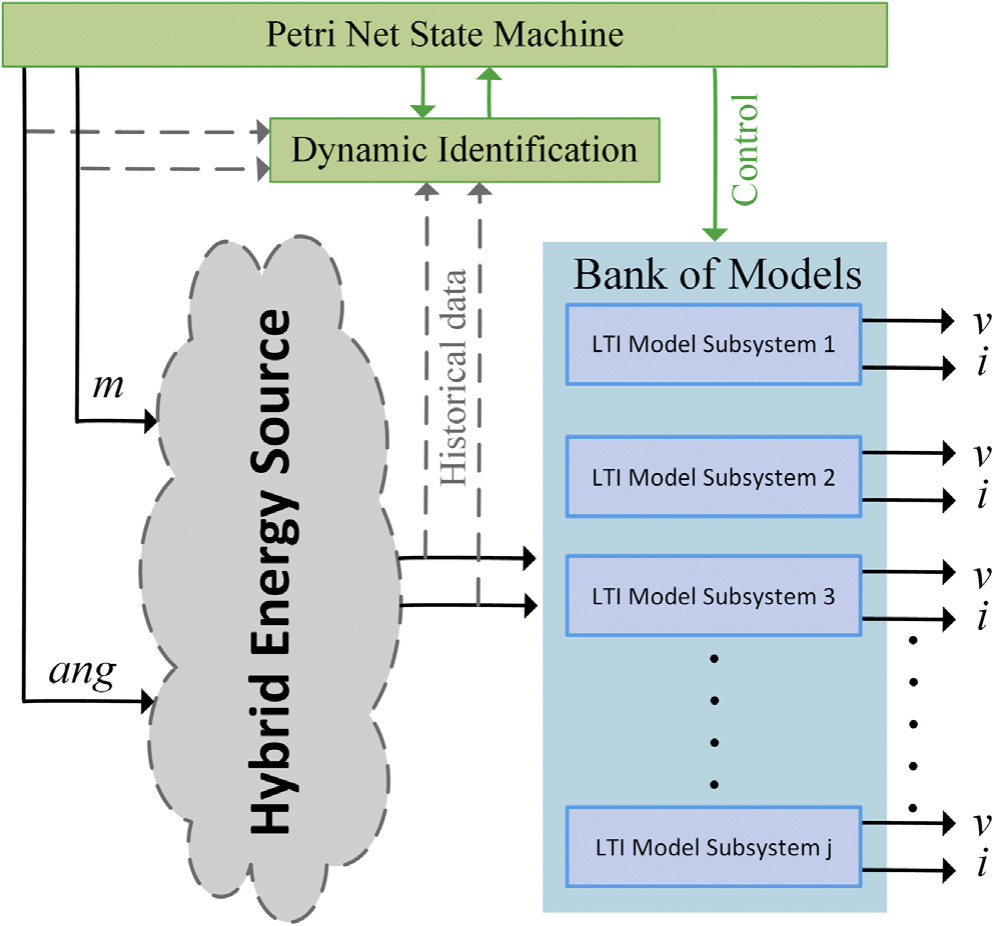
Bank of LTI model.

Figure 5 illustrates the proposed approach that allows obtaining the set of linearized models for the AC/DC HMG. The PN state machine introduces the preset disturbances of the input variable m-index (*m*) and angle (*ang*) to the VSCs, coordinates the identification strategy and the assignment of the mathematical model obtained for each system of the MG in the form of a bank of ordered models. The identification of the models is based on data observed of *m* (*t*), *ang* (*t*), *v* (*t*) and *i* (t).

As can be seen in Figures 10, 11, 12, and 13, the dynamic response of the current (*i*) and voltage (*v*) outputs of a bidirectional DC/AC VSC is non-linear. In itself, MGs are highly non-linear systems where general linear and non-linear models of DG and distribution systems exhibit high mathematical uncertainty and complexity respectively [5].

**Figure 6:**
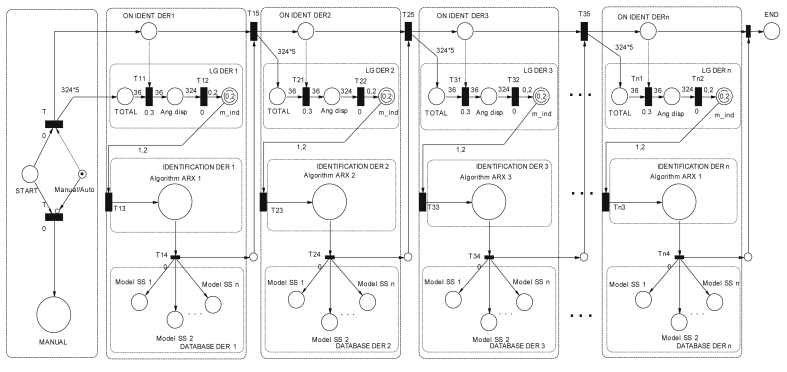
State machine using PN.

Table 1 contains the description of the variables used in the methodology detailed in algorithm 1. The PN coordinates the identification process that is shown in Figure 6.

**Table 1:** Variables of the PN strategy.

Symbology	Variable
*onDer*_*1*_*; onDer*_*n*_	Start status of the methodology for the DG_*n*_.
*total*_*1*_*; total*_*n*_	Test signals buffer for the angle.
*LG*_*d1*_*; LG*_*dn*_	Macro-state of start of test signal injection.
*Md*_*11*_*; Md*_*12*_*Md*_*13*_*; Md*_*21*_*Md*_*22*_*; Md*_*23*_	Data of the input variable j measure of the plant.
*arx*_*1*_*; arx*_*n*_	Macro state of start of the Identification algorithm for DG_*n*_*.*
*next*_*1*_*; next*_*n*_	Start status of the process in the DG_*n+1*_.

**Figure 7:**
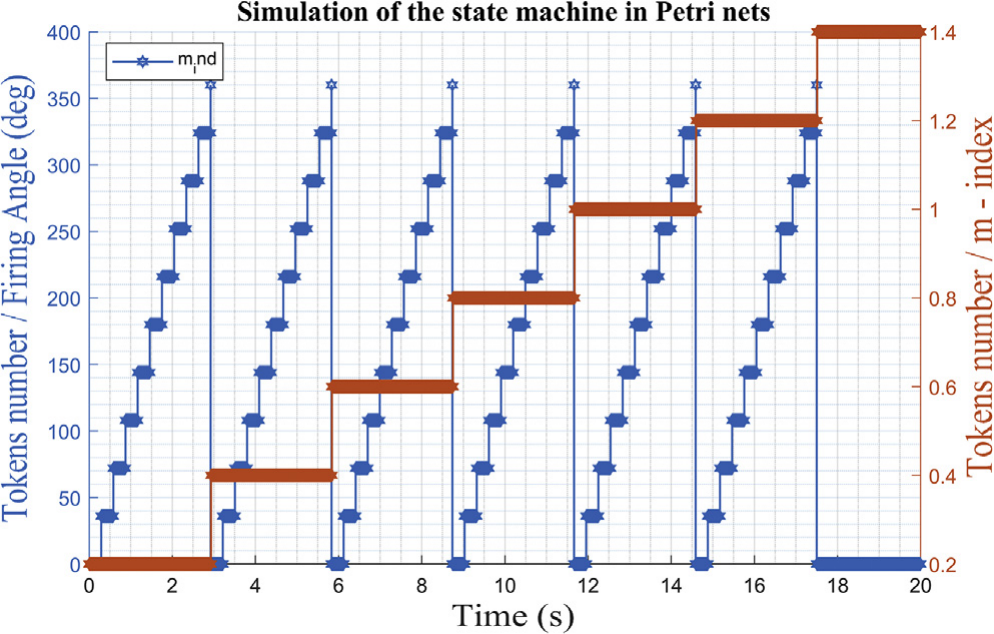
Test signal and setpoint for bi-directional AC/DC converters, inverters or rectifiers.

The strategy based on PN (Figure 6) models a state machine that allows the introduction of a series of type test signals (Figure 5), to coordinate the identification algorithm with the observed data between the different systems, and organizes the bank of linearized models for different operating points in the various systems that make up the MG. Figure 7 shows the test signals created by the strategy. The bank of LTI models obtained is accurate enough to adopt methodologies, control strategies, make hierarchical decisions about a MG under study, and even design strategies to estimate states.

Algorithm 1 PN model methodology for system test.

**Table:** 

Step 1: Set up the distribution of initial Tokens for the PN model, M_o_.
Step 2: Determine the characteristic vector, Ui for the transition state as a fault state.
Step 3: Construct the incidence matrix, C (P,T) of the PN.
Step 4: Determine the dynamic transition process model through vector M_1 ._
Step 5: Using the vector M_1_ redistribute the token after the first shot start.

The use of the step type test signal generated by the PN state machine allows obtaining the variables that feed the identification algorithm. A main advantage is its simplicity, in addition to clearly showing the non-linearity of the system. On the other hand, due to the non-linearity of the MG and its components, the use of this type of test signals has as a main disadvantage that it introduces a rather large variation between the stable operating points of the system and prevents the exact identification between a state and another.

If the developed state machine must be updated because models are required to identify new variables, or the objectives change according to a new problem, the PN update can be carried out without major complexity thanks to its graphic design.

**Figure 8:**
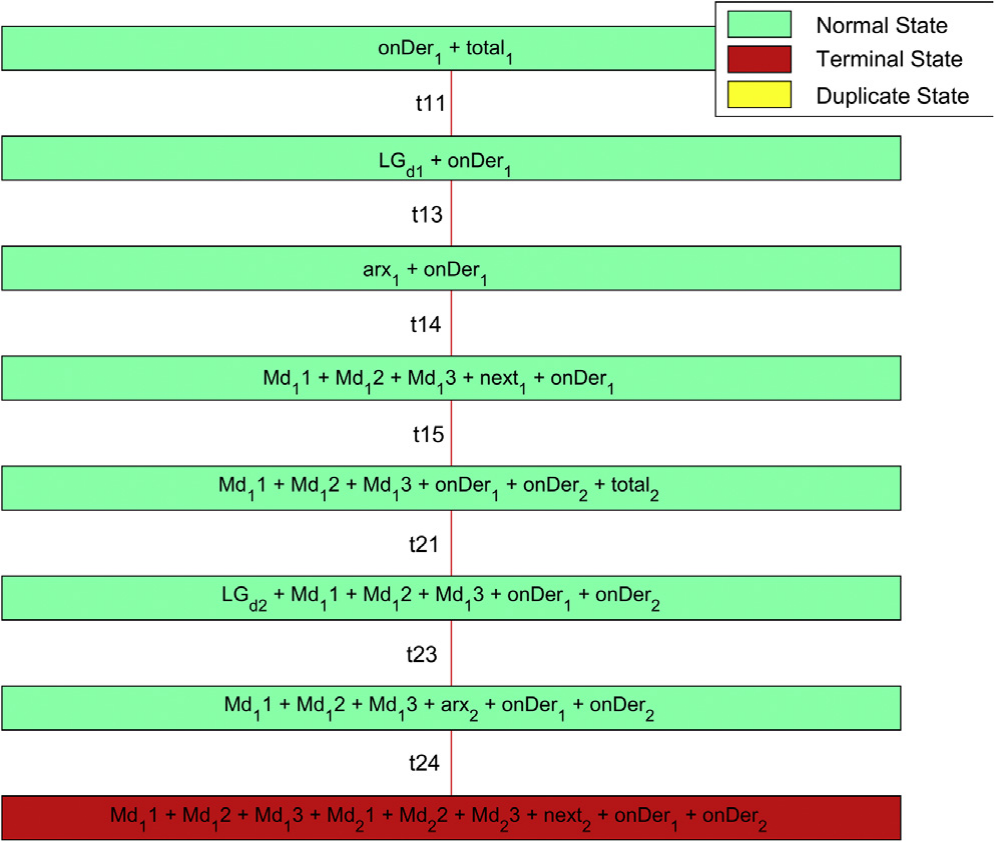
PN coverage tree designed.

Figure 8 shows the coverage tree [39] on ident *DER*n in the implemented strategy. If the coverability tree of the PN is analized, the state machine can be implemented in any computing device [46].

### 5.1 Identification algorithm

The state machine based on PN is complemented by an identification algorithm using ARX (see Table 2 and algorithm 2) that can freely determine the order of the model based on the observed variables of inputs and outputs. In Step 1, the algorithm reads the sensed data after the sampling stage. Then proceeds with the analysis of the data and its visualization before proceeding to evaluate the problem. Finally, the identification is done and the model is converted to continuous time [47].

**Table 2:** Variables.

Symbology	Variable	Unit
*n*	Plant identification number.	u
*Ys*_*j*_	Data of the output variable *i* measurement of the plant.	[V, A]
*Ue*_*i*_	Data of the input variable j measurement of the plant.	[u, degrees]
*t*	Time.	s
*sys*	Mathematical model of the plant in state space.	na
*e*_*i*_	Identification error of the output variable *i*.	%
*u*_*o*_	Initial input conditions	[u, degrees]
*y*_*o*_	Initial output conditions	[u, degrees]

Once the model in state space is obtained in Step 6, it is followed by the simulation of the response in continuous time for the dynamic system with arbitrary inputs, and the estimation error is calculated.

Algorithm 2 Obtaining the dynamic model of the plant *n*.

**Table:** 

Step 1: **Input**: {Ys_j_ (t), U_ei_ (t), u_o_, y_o_,t} ∈ R;
Step 2: **Output**: {*e*_*i*_ (*t*), *A*, *B, C, D*} *∈ R*; ^*sys*;^
Step 3: **Initialize:***A =* [0 0 … 0; 0 0…0]; *B =* [0 0 … 0; 0 0 … 0]; *C =* [0 0 … 0; 0 0 … 0]; *D =* [0 0… 0; 0 0 … 0]; *na* = [2 2 … 2; 2 2 … 2]; *nb* = [2 2… 2; 2 2 …2]; *nk* = [0 0 …0; 0 0 … 0]; *Ys*_*i*_*= Y*_*i*_ (*data*)*; Ue*_*j*_*= U*_*j*_ (*data*)*; t = Ys* (*time*); *n = longitud* (*Ys*_*i*_); *Data =* [*Ys*_*i*_*- y*_*o*_*Ue*_*j*_*- u*_*o*_]; *T = t* (*2*) *– t* (*1*)*;* *Nn* = [*na nb nk*];
Step 4: *sys* ← ARX (*Data*; *nn*); *sys ← Sett (sys,T);*
Step 5: *sys_c ← d2c (sys,'tustin')*;
Step 6: {*A, B, C, D*} ← *ssdata* (*tc*); s*ys_ss = ss* {*A, B, C, D*};
Step 7: *y'*_*s*i_ ← **simulate** s*ys_ss* (*ft*, *u*, *t*);
Step 8: *e*_*j*_ = prom (abs (*y'*_*s*i_ (*data*) – *Y*_*si*_)); **if***e*_*j*_*> 0.1* **go to** Step *3;***endif;**
Step 9: **Return**: s*ys_ss*^;^*A*; *B*; *C*; *D*; *e*_*j*_;

## 6 Case study

This section presents the results of the proposed automatic identification method using a benchmark model of an AC/DC HMG presented in [7]. This model is simulated in Matlab/Simulink environment.

This AC/DC HMG includes:

• One diesel generator,
• Two photovoltaic (PV) systems,
• Two battery energy storage system,
• Various linear and non-linear loads.

**Figure 9:**
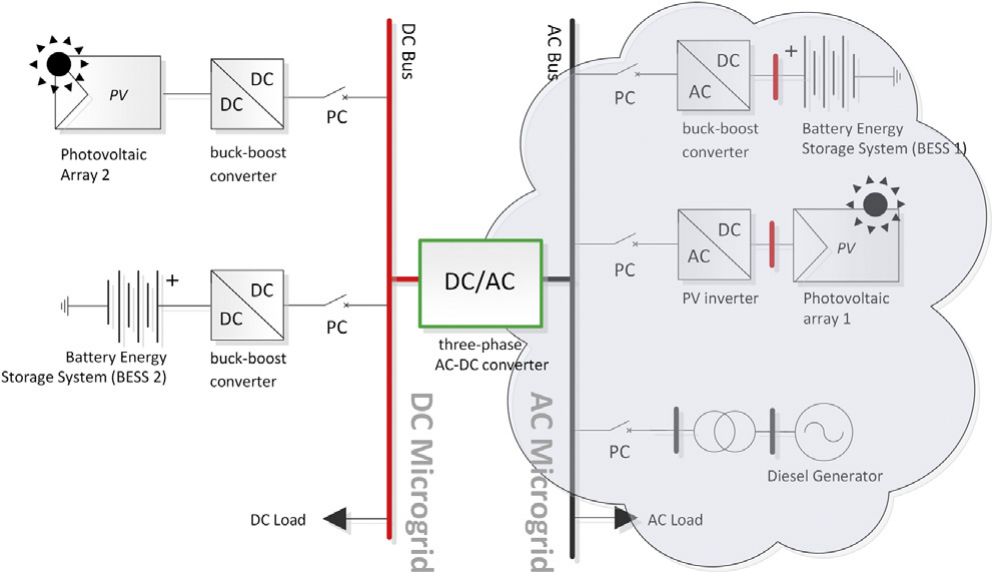
Diagram of AC/DC hybrid microgrid case study.

**Figure 10:**
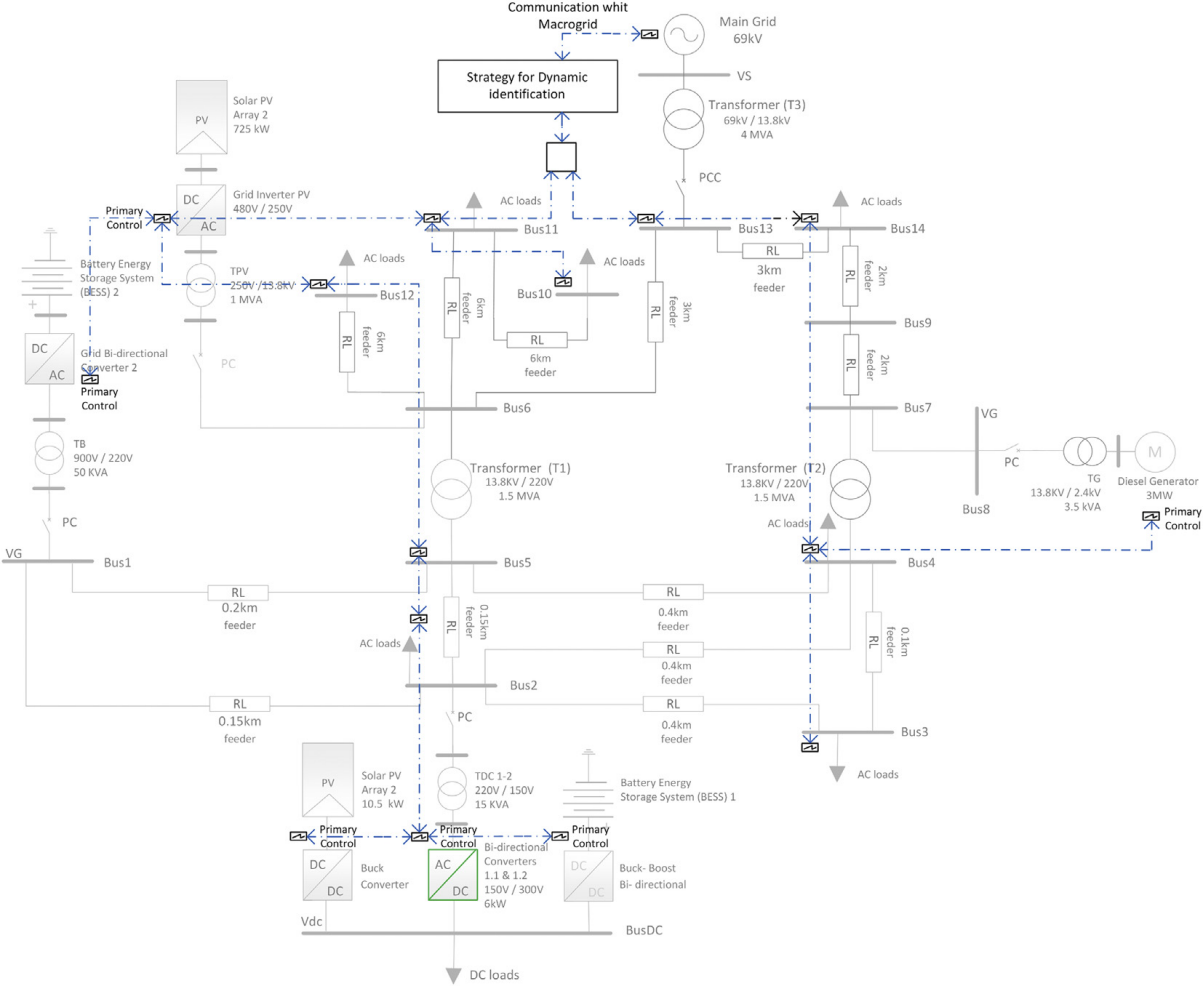
Test system single-line diagram of AC/DC hybrid microgrid.

The results shown in this article are related to the bidirectional AC/DC inverter shown in green color in Figures 9 and 10.

This AC/DC HMG has two AC voltage distribution levels (the primary level is 13,8 kV and the secondary level is 220 V) and one DC distribution level (300V). The AC MG operates at a frequency of 60 Hz. The DC busbar comprises of the BESS 2 and PV Array 2. The Array 1 has 42 modules which develops a nominal power of 10,5 kW whereas Array 2 has 1750 modules, which develops a nominal power of 725 kW. Finally, both arrays operate with a total solar irradiance of G = 1000 W/m^2^ and a temperature of 25 °C [7].

The BESS that operates in the DC bus and consists of 1 lithium-ion battery unit of 120 VDC nominal voltage. The BESS that operates near LV AC bus consists of 3 nickel-metal-hydride (Ni–MH) battery units of 650 VDC nominal voltage. The parallel-connected batteries are connected to an interfaced inverter in a cascaded topology. The first BESS system is connected through a boost-buck bidirectional converter, while a PV Array is connected to the DC link through a boost converter. The DC bus is linked to the AC MG through two parallel bidirectional converters which can operate as rectifiers or inverters [7].

This is designed to convert the 300V DC voltage to an output voltage of 150 V AC that permits to deliver the excess power generated in the DC bus. For instance, if power is needed in the DC bus for lack of power in the PV and the BESS 1, the system will convert the 150V AC voltage to a DC output voltage of 300V. The increase of robustness, flexibility, and performance obtained by the DC MG is an important solution which allows a maximum performance for a wide range of power [7].

### 6.1 Observed data for three operating points of the DC bus

**Figure 11:**
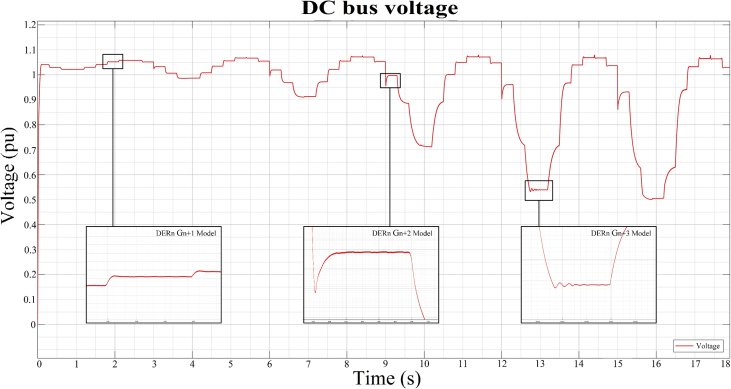
Observed voltage data on the DC MG bus.

**Figure 12:**
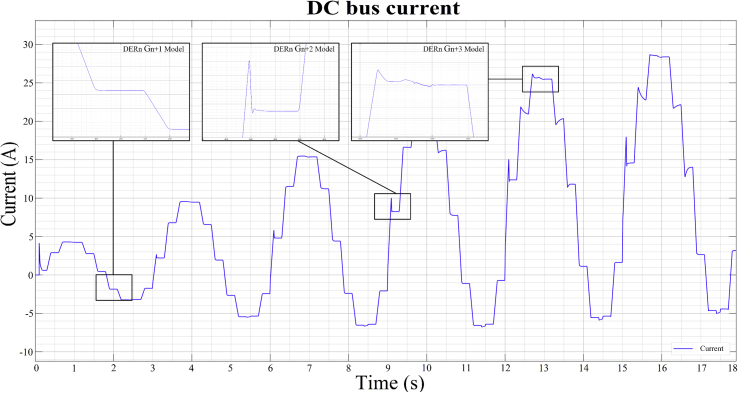
Observed current data on the DC MG bus.

In order to be able to show the results in this investigation, three random operating points must be selected (see Table 3), two in inverter mode corresponding to the observed data *DER_n_ Gn+2* and *DER_n_ Gn+3*; and another in rectifier mode corresponding to *DER_n_ G_n+1_.* These stable operating points are then simulated to obtain the dynamic models that correspond to the states previously mentioned. The opted models are calculated and stored by the proposed state machine, forming a bank of LTI models. For this case of study, the identification of the dynamic model of the bidirectional AC/DC VSC (*DER*_*n*_ *G*_*n*_) between the DC MG and the AC MG is made. The operating points are selected to identify the models presented in Table 3, Figures 11 and 12. These two figures show the dynamic behavior of the variables of voltage and current for the test signal imposed on Figure 7.

**Table 3:** Test signals adjusted to establish operating points on the DC MG bus.

Operating Point	Step signals and **Initial input conditions**		Initial output conditions, V (pu), I (A)	
	M	ang	y_o__V	y_o__i
*DER*_*n*_*G*_*n+1*_	**0**–0.2	**180**^**o**^ – 216^o^	1.044	0.0136
*DER*_*n*_*G*_*n+2*_	**0.6**–0.8	**324**^**o**^ – 0^o^	1.053	-2.085
*DER*_*n*_*G*_*n+3*_	**0.8**–1	**72**^**o**^ – 108^o^	0.705	20.8

#### 6.2 Observed data for three operating points of the AC bus

This section shows the results of the proposed method for the bidirectional AC/DC converter in its inverter mode. In addition, the dynamic responses of the voltage and current in phases *a, b* and *c* for the imposed test signal are shown (Figure 7: *m* and *ang*). Subsequently, the operating point corresponding to *DER_n_ Gn+3* is selected.

**Figure 13:**
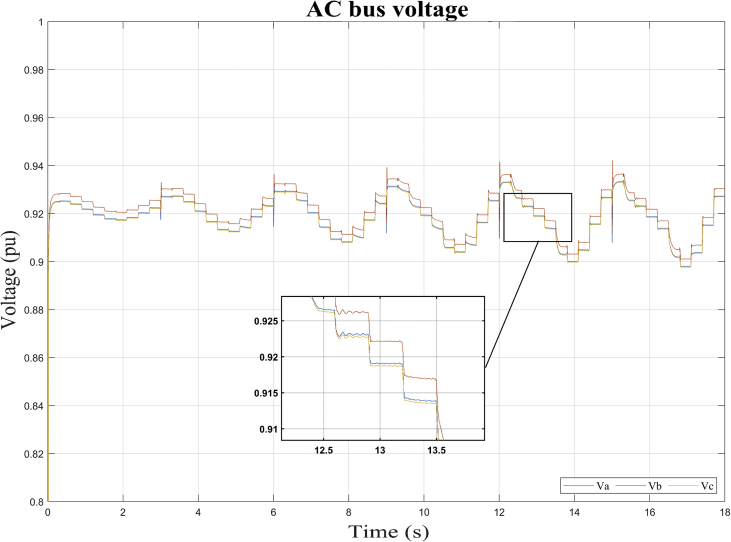
Observed voltage data on the AC MG bus.

**Figure 14:**
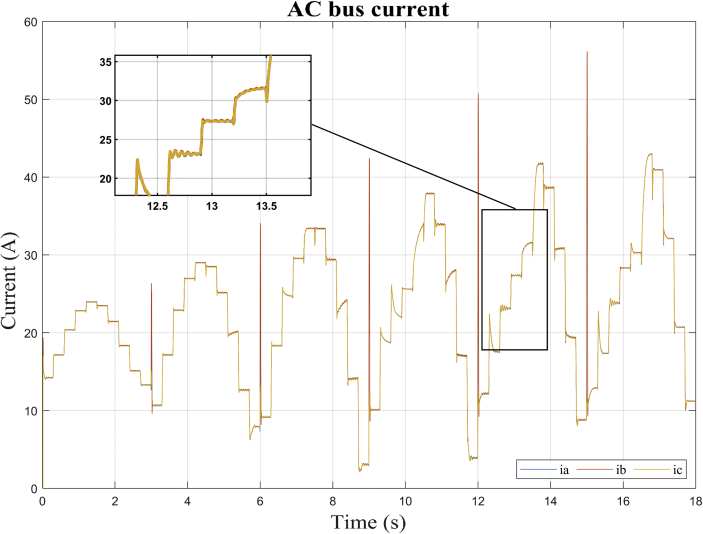
Observed current data on the AC MG bus.

In this case, the dynamic model is identified for the bidirectional AC/DC VSC coupling (*DER_n_ G*_*n*_) between the DC MG and the AC MG. The operating points selected to carry out the study are also shown. It is apparent from Figures 13 and 14 the dynamic behavior of the variables of voltage and current for the test signal imposed on Figure 7. The observed data are selected for operating point *DER_n_ Gn+3* (see Table 4) and later the system is identified.

**Table 4:** Test signal adjusted to establish operating point on the AC microgrid bus.

Operating Point	Step signas		Initial output conditions, V (pu), I (A)					
	m	ang	Va	Vb	Vc	ia	ib	ic
*DER*_*n*_*G*_*n+4*_	0.8	108^o^	0.922	0.925	0.922	26.46	26.49	26.4

## 7 Analysis of results for the identification strategy on the DC/AC microgrid

Once the PN algorithm assigns the mark to the macro-state of system identification (Bidirectional AC/DC converter), the implemented identification algorithm based on ARX method is automatically executed. Next, the algorithm performs the analysis and adjustment of the data obtained after applying the step type test signal between two operating points.

### 7.1 Identification and analysis on the DC bus for the tree operating points

**Figure 15:**
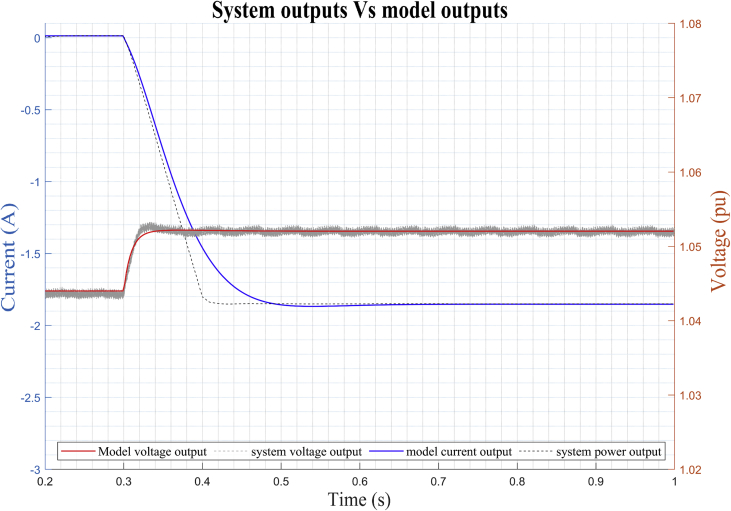
Dynamic response of DER_n_ G_n+1_ model versus observed data.

After the identification process is carried out by the algorithm, the obtained model is simulated and the results are compared with the observed data of the outputs (*V*_DC_ (*t*) and *i*_DC_(*t*)) for ‘*DER_n_ G_n+1_*’ (see Figure 15). Then, the method performs the identification using the data obtained around the corresponding stable operating point *DER_n_ G_n+1_* and we obtain a MIMO system is obtained with two inputs (*m* and *ang*) and two outputs (*V*_DC_ and *i*_DC_) for dynamic model of the bidirectional AC/DC VSC, such that:

(16) $$U_{ei}\left(t\right)\;=\;\left[m\;\left(t\right)\;ang\;\left(t\right)\;\right];\;$$

(17) $$Y_{si}\left(t\right)\;=\;\left[V_{DC}\;\left(t\right)\;\;\;i_{DC}\left(t\right)\;\right];$$

As detailed in Figure 15, there is a high degree of correlation not only between the results of the mathematical model in the state space obtained, but also with the results of the experimental study of the bidirectional AC/DC VSC responsible for the flow of electrical energy between the MG in DC and AC of the case of study.

Verification was done by means of numerical simulations carried out in Matlab in order to show the effectiveness of algorithm 1 and the obtained models of discrete state space.

Estimated parameters of the stable operating point *DER_n_ G_n+1_*:

(18) $$A=\left[\begin{array}{cccc} -577.735 & -1.379 & 1.461 & 1.379\\ 169.707 & 421.398 & -169.707 & 461.809\\ 368.751 & 1.175 & -1251.958 & -1.175\\ -313.386 & -422.166 & 313.386 & -461.041 \end{array}\right]$$

(19) $$B=\left[\begin{array}{cc} 0 & 0.0356\\ 0 & -0.3137\\ 0 & -0.0053\\ 0 & 0.2728 \end{array}\right]$$

(20) $$C=\left[\begin{array}{cccc} 1.654 & 0.0016 & \mathrm{-1}\mathrm{.6541} & \mathrm{-0}\mathrm{.0016}\\ \mathrm{-0}\mathrm{.1921} & 0.5229 & 0\mathrm{.1921} & \mathrm{-0}\mathrm{.5229} \end{array}\right]$$

(21) $$D=\left[\begin{array}{cc} 0 & -2.1458e-5\\ 0 & -3.3064e-4 \end{array}\right]$$

**Figure 16:**
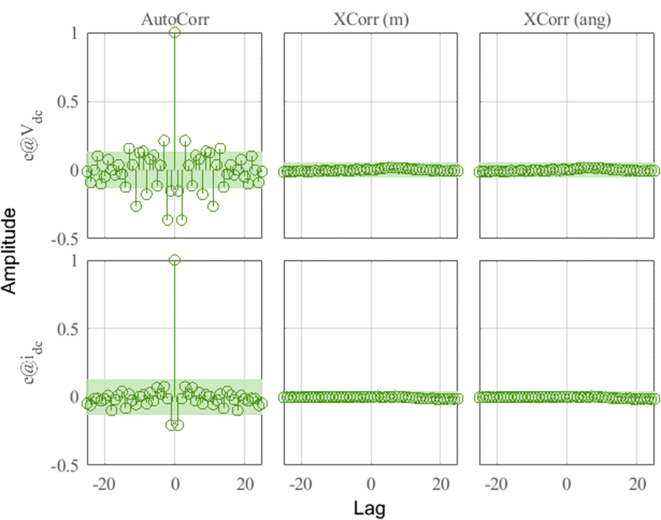
Residue correlation of identification input and output data for DER_n_ G_n+1_ model.

In Figure 16, the plots of the correlation of the outputs of the model with the HMG data can be observed for the different outputs of the system. Correlations are calculated until the maximum lag of 25%. The green shaded region around the X-axis marks a 99% confidence level for statistically non-significant correlations.

**Figure 17:**
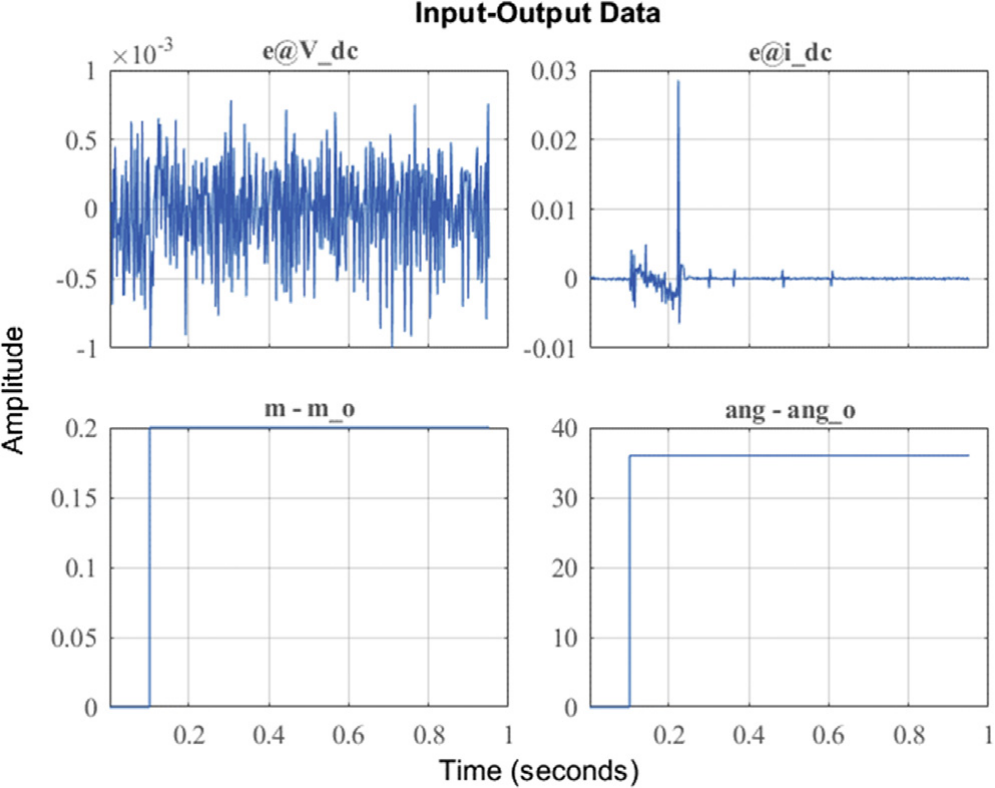
Identification errors for DER_n_ G_n+1_ model.

In Figure 17, the dynamics of the identification errors (*e*) of the outputs *V*_dc_ and *i*_dc_ can be observed and analyzed. It shows the autocorrelations of the residues and the cross correlation between residues and inputs. By observing the dynamics of the residual inputs in the figure, the effectiveness of the ARX algorithm for *DER_n_ G_n+1_* can be evaluated in more detail.

The results obtained for the *DER_n_ Gn+2* and *DER_n_ Gn+3* operating points shown below evidence the high correlation between the results of the mathematical model in the state space and the experimental study of the converter.

**Figure 18:**
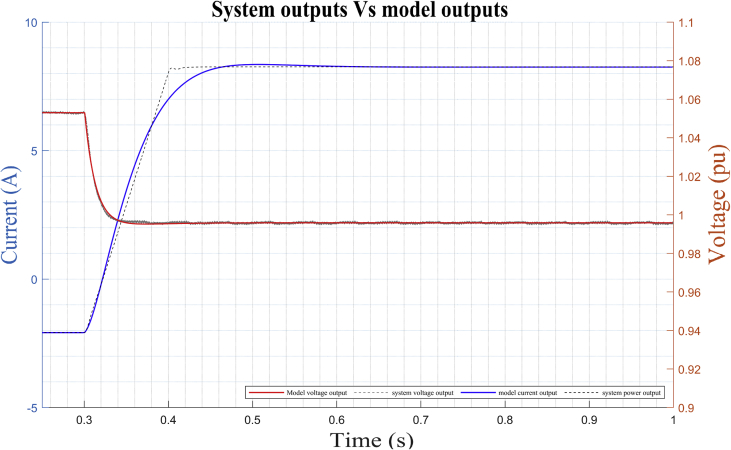
Dynamic response of DER_n_ Gn+2 model versus observed data.

The estimated parameters of the stable operating point *DER_n_ Gn∖2* are (see dynamic response of the model in Figure 18):

(22) $$A=\left[\begin{array}{llll} -0.0834e3 & -0.0003e3 & 1.1484e3 & 0.0003e3\\ 2.3430e3 & 0.5139e3 & -2.3430e3 & 0.5511e3\\ 0.0029e3 & 0.0003e3 & -1.0679e3 & -0.0003e3\\ -1.8073e3 & -0.5143e3 & 1.8073e3 & -0.5507e3 \end{array}\right]$$

(23) $$B=\left[\begin{array}{cc} 0 & -0.0139\\ 0 & -0.1209\\ 0 & -0.0000\\ 0 & -0.0036 \end{array}\right]$$

(24) $$C=\left[\begin{array}{llll} 1.0783 & 0.0003 & -1.0783 & -0.0003\\ -2.2000 & 0.5175 & 2.2000 & -0.5175 \end{array}\right]$$

(25) $$D=\left[\begin{array}{cc} 0 & -0.0850e-4\\ 0 & 0.3531e-4 \end{array}\right]$$

**Figure 19:**
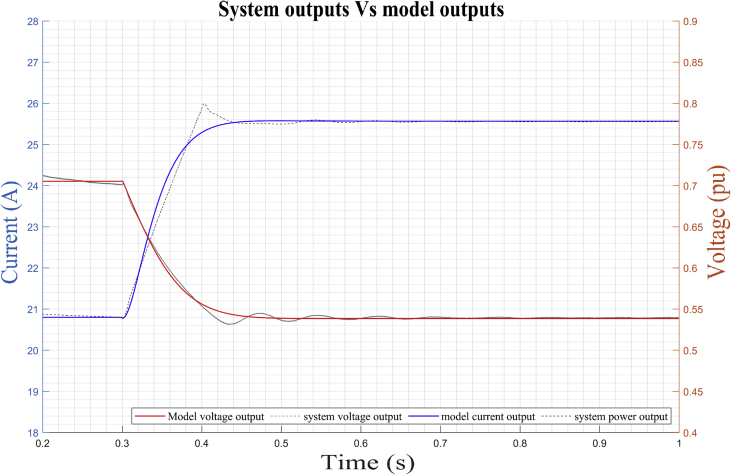
Dynamic response of DER_n_ Gn+3 model versus observed data.

The estimated parameters of the stable operating point DER_n_ Gn∖xFE3 are (see dynamic response of the model in Figure 19).

(26) $$A\;=\;\left[\begin{array}{cccc} 0.0054e3 & -0.0040e3 & 1.0915e3 & 0.0040e3\\ 0.7903e3 & 0.5075e3 & -0.7903e3 & 0.5894e3\\ -0.0419e3 & 0.0034e3 & -1.0550e3 & -0.0034e3\\ -0.7164e3 & -0.5081e3 & 0.7164e3 & -0.5888e3 \end{array}\right]$$

(27) $$B=\left[\begin{array}{cc} 0 & -0.0616\\ 0 & 0.0616\\ 0 & -0.0056\\ 0 & 0.4890 \end{array}\right]$$

(28) $$C=\left[\begin{array}{cccc} 0.9950 & 0.0036 & -0.9950 & -0.0036\\ -0.7204 & 0.5373 & 0.7204 & -0.5373 \end{array}\right]$$

### 7.2 Identification and analysis on the AC for the operating point

**Figure 20:**
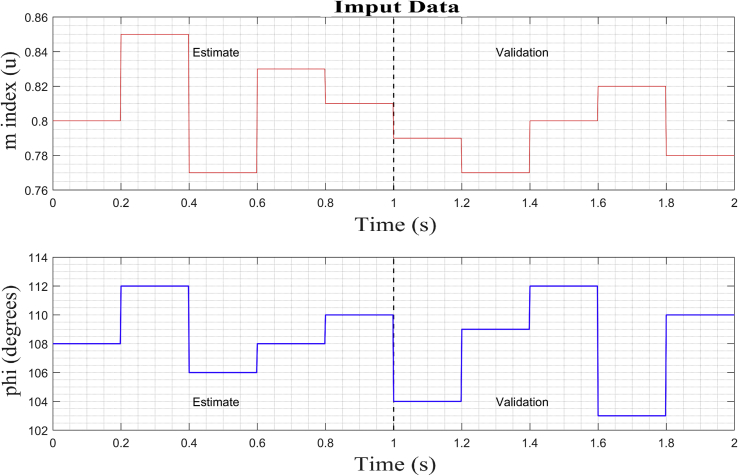
Test signal and setpoint for operating point on AC inverter.

The PN state machine injects test signals typically staggered (Figure 20) and collects the data of the outputs of *V*_abc_ and *i*_abc_. From the data set observed divided into two parts, a separate one was used to estimate the linear model on the operating point. Then, the second part of the data was used with the objective of cross validation and to be able to evaluate the adjustment criteria of the ARX algorithm.

Then the method performs the identification using the data obtained around the corresponding stable operating point *DER_n_ Gn+4* and we obtain a MIMO system is obtained with two inputs (*m* and *ang*) and six outputs (*V*_a_, *V*_b_, *B*_c_, *i*_a_, *i*_b_, and *i*_c_) for dynamic model of the bidirectional AC/DC converter, such that:

(30) $$U_{ei}\left(t\right)\;=\;\left[m\;\left(t\right)\;ang\;\left(t\right)\;\right];\;$$

(31) $$Y_{si}\left(t\right)=\;\left[V_{abc}\;\left(t\right)\;\;\;i_{abc}\left(t\right)\;\right];$$

**Figure 21:**
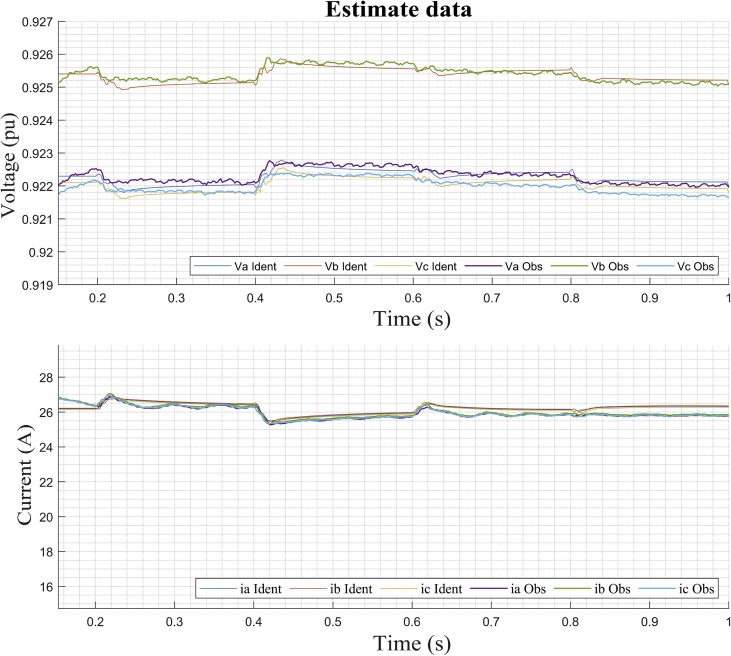
Dynamic voltage response of *DER*_*n*_*G*_*n+4*_ and estimates data.

Figure 21 shows the high degree of similarity between the results obtained by simulating the model mathematical in the linear state space (identified by the ARX structure) and the estimate data observed through the digital simulation of the bidirectional the bidirectional AC/DC VSC for the operating point *DER_n_ Gn+4.*

**Figure 22:**
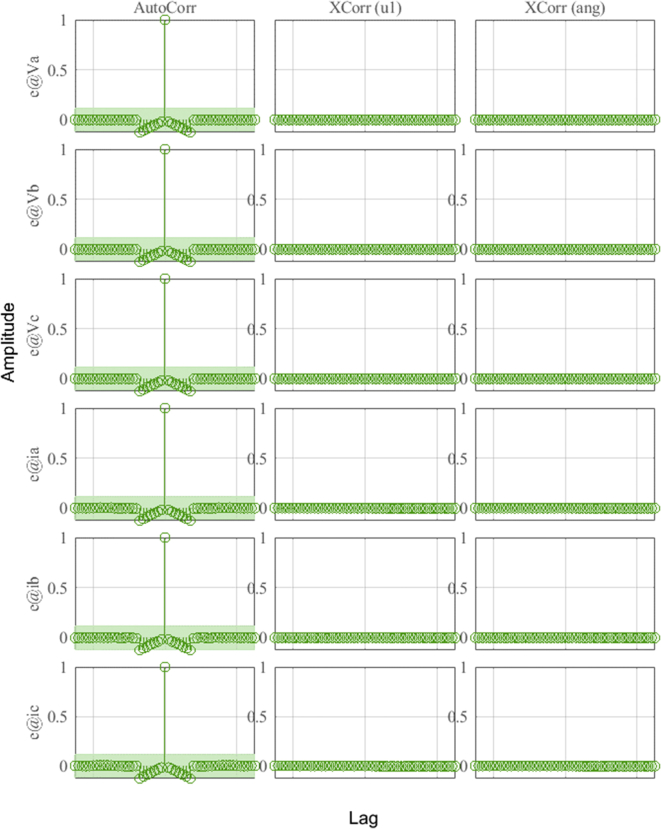
Residue correlation of identification input and output data for DER_n_ Gn+4 model.

**Figure 23:**
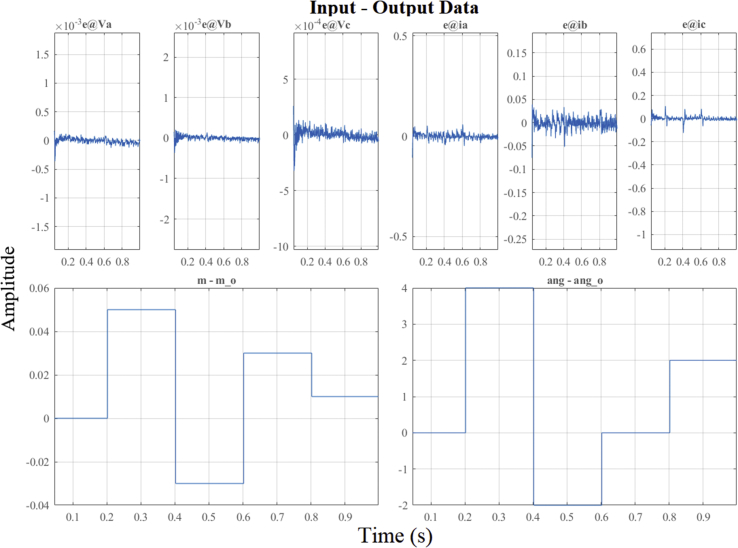
Identification errors for DER_n_ Gn+4 model.

Figure 22 and Figure 23 show the correlation analysis according to the different outputs of the dynamic system and analysis of identification errors (*e*) of the *V*_abc_ and *i*_abc_ outputs using the estimation data.

**Figure. 24:**
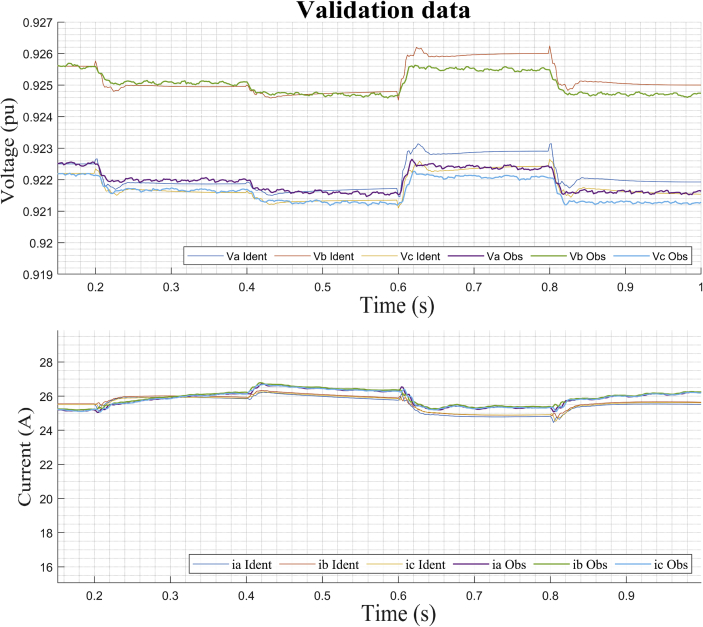
Dynamic current response of *DER*_*n*_*G*_*n+4*_ and validation data.

Figure 24 show the high degree of similarity between the results of the mathematical model in state space obtained under digital simulation with the validation data from the simulated experimental study of bidirectional AC/DC VSC for the DER_n_ *DER_n_ Gn+4.*

**Figure 25:**
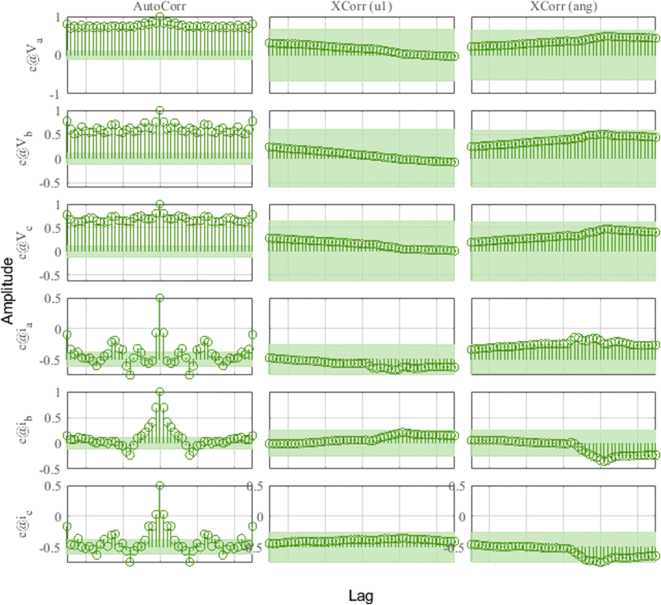
Residue correlation of validation input and output data for DER_n_ Gn+4 model.

**Figure 26:**
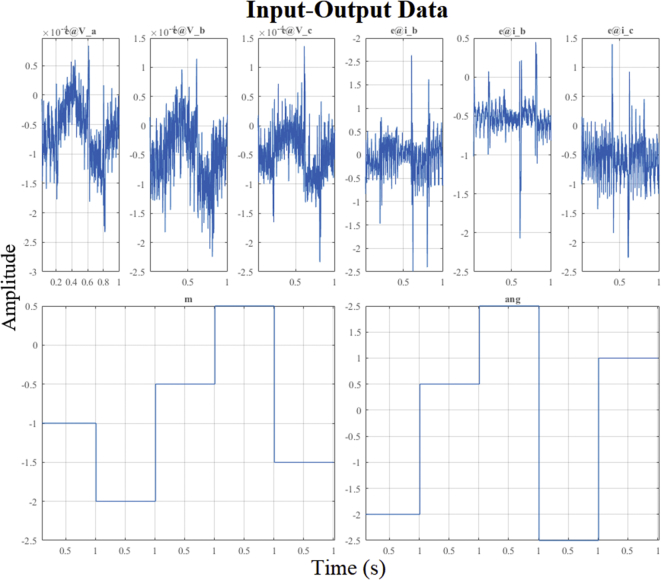
Identification errors for DER_n_ Gn+4 model.

Figure 25 and Figure 26 show the correlation test analysis according to the different outputs of the dynamic system and analysis of identification errors (*e*) of the *V*_abc_ and *i*_abc_ outputs using the validation data.

The results show that the bank of models (in the various operating points) can reproduce the non-linear dynamic characteristics of the HMG elements with precision. Thanks to the simplicity of the method and the bank, this can be used for the development of techniques such as: Fault Detection and Identification, Fault Tolerance, State Estimation among others.

For implemented MG applications, the different signals of inputs and outputs of the dynamic system should be captured and characterized with the goal of adding phenomenological information about the plant. This would allow a process of more precise identification of the models that make up the bank and may later function as a robust general model for all the permitted operating points. Based on the results achieved, the strategy is characterized by the balance between the precision of the estimation accuracy and linearization of the identified models.

The estimated parameters of the operating point *DER_n_ Gn+4* are:

(32) $$A\;=\;[\begin{array}{cccc} 0.0052e4 & 0.0063e4 & -0.0178e4 & 0.0167\\ -0.0544e4 & -0.0117e4 & 0.0604e4 & 0.4058\\ 0.0191e4 & -0.0532e4 & 0.0282e4 & 0.2786\\ 1.4599e4 & 4.9448e4 & -7.7685e4 & 0.0342e4\\ 2.9748e4 & -5.1970e4 & 0.9437e4 & -0.0150e4\\ -1.1492e4 & -3.4439e4 & 3.5174e4 & 0.0366e4\\ -0.0281e4 & 0.0295e4 & -0.0018e4 & -0.1702\\ 0.0218e4 & 0.0375e4 & -0.0603e4 & -0.7634\\ -0.0237e4 & 0.0444e4 & -0.0212e4 & -0.2182\\ 3.4012e4 & -9.5517e4 & 7.4167e4 & -0.0229e4\\ -4.3880e4 & 0.6821e4 & 4.8203e4 & 96.8106\\ 6.3117e4 & -0.6109e4 & -4.7140e4 & 0.0253e4 \end{array}......\begin{array}{cccc} -0.1783 & -0.0121 & 0.0948e4 & -0.0063e4\\ -0.7254 & 0.1279 & 0.0544e4 & 0.1117e4\\ -0.3416 & -0.0571 & -0.0191e4 & 0.0532e4\\ 0.0450e4 & -0.0320e4 & -1.4599e4 & -4.9448e4\\ 0.0321e4 & 0.0276e4 & -2.9748e4 & 5.1970e4\\ -0.0350e4 & 0.0453e4 & 1.1492e4 & 3.4439e4\\ 0.3492 & -0.0337 & -0.0719e4 & -0.0295e4\\ 0.8945 & 0.0215 & -0.0218e4 & -0.1375e4\\ 0.2859 & 0.0271 & 0.0237e4 & -0.0444e4\\ -0.0463e4 & 0.0222e4 & -3.4012e4 & 9.5517e4\\ -0.0446e4 & -0.0110e4 & 4.3880e4 & -0.6821e4\\ 0.0229e4 & -0.0441e4 & -6.3117e4 & 0.6109e4 \end{array}......\begin{array}{cccc} 0.0178e4 & -0.0167 & 0.1783 & 0.0121\\ -0.060e4 & -0.4058 & 0.7254 & -0.1279\\ 0.0718e4 & -02786 & 0.3416 & 0.0571\\ 7.7685e4 & 0.0658e4 & -0.0450e4 & 0.0320e4\\ -0.9437e4 & 0.0150e4 & 0.0679e4 & -0.0276e4\\ -3.5174e4 & -0.0366e4 & 0.0350e4 & 0.0549e4\\ 0.0018e4 & 0.1702 & -0.3492 & 0.0337\\ 0.0603e4 & 0.7634 & -0.8945 & -0.0215\\ -0.0788e4 & 0.2182 & -0.2859 & -0.0271\\ -7.4167e4 & -0.0771e4 & 0.0463e4 & -0.0222e4\\ -4.8203e4 & -0.0100e4 & -0.0554e4 & 0.0110e4\\ 4.7140e4 & 0.0253e4 & -0.0224e4 & -0.0559e4 \end{array}]$$

(33) $$B=\left[\begin{array}{cc} 0.2470 & -0.0044\\ 0.2796 & -0.0068\\ 0.0218 & -0.0027\\ 235.2779 & -2.3631\\ -20.3837 & -0.0492\\ 20.3973 & 1.4566\\ 0.1942 & -0.0033\\ 0.0949 & -0.0018\\ 0.2089 & -0.0032\\ -185.8761 & 2.4476\\ -69.2523 & 1.2198\\ -36.3375 & 0.0299 \end{array}\right]$$

(34) $$C=[\begin{array}{cccc} 0.9483 & -0.0631 & 0.1780 & -1.6723e-5\\ 0.5436 & 1.1169 & -0.6037 & -0.0004\\ -0.1913 & 0.5316 & 0.7180 & -0.0003\\ -14.5989 & -49.4478 & 77.6854 & 0.6578\\ -29.7482 & 51.9697 & -9.4374 & 0.1504\\ 11.4916 & 34.4388 & -35.1744 & -0.3665 \end{array}......\begin{array}{cccc} 0.0002 & 1.2089e-5 & -0.9483 & 0.0631\\ 0.0007 & -0.0001 & -0.5436 & -1.1169\\ 0.0003 & 0.0001 & 0.1913 & -0.5316\\ -0.4500 & 0.3204 & 14.5989 & 49.4478\\ 0.6793 & -0.2762 & 29.7482 & -51.9697\\ 0.3500 & 0.5486 & -11.4916 & -34.4388 \end{array}......\begin{array}{cccc} -0.1780 & 1.6723e-5 & -0.0002 & -1.2089e-5\\ 0.6037 & 0.0004 & -0.0007 & 0.0001\\ -0.7180 & 0.0003 & -0.0003 & -0.0001\\ -77.6854 & -0.6578 & 0.4500 & -0.3204\\ 9.4374 & -0.1504 & -0.6793 & 0.2762\\ 35.1744 & 0.3665 & -0.3500 & -0.5486 \end{array}]$$

(35) $$D=\left[\begin{array}{cc} -0.0007 & 0\\ -0.0002 & 0\\ -0.0002 & 0\\ 0.0319 & 0.0008\\ 0.1890 & -0.0028\\ 0.0130 & -0.0007 \end{array}\right]$$

## 8 Future work and areas of research

In future studies where the very fast dynamics and non-linear behavior of MG systems are considered, it will be necessary to complement the strategy with non-linear algorithms such as the Non-linear Autoregressive Exogenous (NARX) model. It also opens the doors for several lines of future research such as: online identification strategies, the study and design of predictive control strategies, adaptive control strategies, robust control strategies, FTCS among others. Finally, other strategies can be designed with the aim of coordinating the automatic and optimal use of the model bank obtained and performing the tuning and online reconfiguration of controllers.

## 9 Conclusions

This paper has considered the use of PNs and ARX algorithms with the aim of developing a hybrid and novel strategy that allows the dynamic identification of HMGs. In addition, the proposal obtains a bank of linearized models in space state that represent the AC/DC HMG. These models are achieved from the different systems that integrate the HMG benchmark model, such as: VSCs (unidirectional or bidirectional), BESSs, PVs, DiGs among others.

Due to the simplicity of the bank of linearized models obtained from the methodology, less computational effort is required for simulations or for the design of the primary and secondary control systems. Additionally, the dynamic behavior of the HMGs AC or DC power elements (including loop control) is represented by the bank of linear models without losing the accuracy of the data observed.

Unlike other proposed methodologies, the use of PNs operate based on a robust mathematical representation while presenting an easy-to-interpret graphic design. The proposed PN model achieves the coordination of the network for the injection of test signals, collection of the observed data, and the coordination of the identification method by ARX. Thus, this methodology not only provides the models for both the DC MG and AC MG, but also coordinates and creates bank of models organized in different modes: isolated, connected, or charge/discharge of BESS systems.

## Declarations

### Author contribution statement

Leony Ortiz, Luis B.Gutiérrez, Jorge W.González: Conceived and designed the experiments; Performed the experiments; Analyzed and interpreted the data; Contributed reagents, materials, analysis tools or data; Wrote the paper.

Alexander Águila: Performed the experiments; Analyzed and interpreted the data; Contributed reagents, materials, analysis tools or data; Wrote the paper.

### Funding statement

This research did not receive any specific grant from funding agencies in the public, commercial, or not-for-profit sectors.

### Competing interest statement

The authors declare no conflict of interest.

### Additional information

No additional information is available for this paper.
